# Beyond the Choice of What You Put in Your Mouth: A Systematic Mapping Review of Veganism and Vegan Identity

**DOI:** 10.3389/fpsyg.2022.848434

**Published:** 2022-06-10

**Authors:** Sara Vestergren, Mete Sefa Uysal

**Affiliations:** ^1^School of Psychology, Keele University, Newcastle upon Tyne, United Kingdom; ^2^Department of Social Psychology, Friedrich Schiller University Jena, Jena, Germany

**Keywords:** vegan, veganism, identity, activism, social movement

## Abstract

In recent years, and in the current climate crisis, the interest in veganism and sustainable diet/lifestyle has increased. This growing interest can also be seen within academia. Therefore, we set out to systematically document and organize the social psychological literature on veganism and vegan identity to identify where the field currently is, and what we need to do next. Following PRISMA guidelines we identified a data set of 26 academic papers published between 2010 and 2021. Through a thematic analysis of the data, we created four categories of study focus and content: (1) vegans as a disadvantaged/stigmatized group, (2) the role of ideology in negative attitudes toward vegans, (3) the role of moral and ethical beliefs in changing or sustaining dietary preferences, and (4) veganism as a social movement and vegan activism. Our analysis emphasizes issues with merging all non-meat eaters, reduction of veganism into dietary or lifestyle choices neglecting the politicized content and movement, lack of processes underlying emergence and endurance of veganism, and decontextualization of vegan identity. What is needed is a more fine-grained exploration that addresses the identified issues to account for the content of vegan identity. This would expand, for example, the motives literature to include and emphasize intersectionality in a vegan identity context. Specifically, to facilitate a more sustainable lifestyle, the content of social dimensions needs to be qualitatively explored.

## Introduction

Veganism is described in various ways by non-vegans, often referring to what vegans do not eat. However, vegans generally refer to veganism as a political philosophy based on the rejection of the commodity status of animals (Pedersen and Staescu, 2014) or as part of an environmentally sustainable ideology (Buttny and Kinefuchi, 2020; Hudepohl, 2021). Although veganism is considered extreme by many people, veganism is gradually becoming a widespread phenomenon not only in western societies but across cultures around the world (Forgrieve, 2018; Jones, 2020). However, among scholars, there is sparse focus on the political, collective, and social movement aspects of veganism. Understanding the wider dimensions of veganism on an ideological and collective level is important in our understanding and application of an environmentally sustainable lifestyle.

Vegan identity can be understood as a shared social identity with rejection of the product-status of animals and the intersectional justice movement against animal exploitation and speciesism as part of identity content (e.g., Tajfel and Turner, 1979). The social identity approach suggests that our group memberships, the social categories we perceive to be part of, make significant contributions to how we see ourselves and our worldview (e.g., Turner et al., 1987). Some social categories and group memberships become salient in our daily lives as we perceive our worldview through that categorization (see Vestergren et al., 2018). Hence, our identification as part of the social category vegans will affect our values and behaviors in all social contexts where appropriate. Consequently, vegan identity and veganism goes beyond the choice of diet (plant-based) to incorporate veganism as an identity characteristic influencing actions and values derived from norms of the social identity. However, whether veganism is part of a social identity, or a social identity in itself is likely to depend on the social context. For example, veganism can be part of social identity content, you might be an animal-rights activist and reject harm to animals in all forms which makes veganism part of your animal-rights identity. Importantly, vegan identity includes social values and norms, and should thereby be seen as more than a dietary choice or identity. What veganism includes might depend on the salient social identity, such as values and behaviors tied to feminism, environmentalism, or animal-rights. Furthermore, veganism is often expressed through actions in relation to others, and not only oneself or one's own group. For example, Judge et al. (2022) emphasize in their Social Identity Model of Vegan Activism (SIMVA) that vegan identity also includes an active component of trying to promote vegan norms to others which goes beyond the food you put in your mouth.

Individuals can have various and diversified motives for becoming vegan including health-related, environmental, animal and social justice. Hence, a definition focused solely on diet does not capture the different levels of veganism (see North et al., 2021). With the transformative nature of vegan identity that goes beyond the vegan diet itself, the motives become converged to political and social justice-oriented aspects of veganism, which reflects the intersectional nature of the movement. It is also important to acknowledge that social identities can develop based on a perception of shared reactions or struggle to/in a situation (e.g., Thomas and McGarty, 2009; Thomas et al., 2012). Hence, the current climate crisis could provide a context where a shared identity around the climate emerges, which contains veganism as a shared value and behavioral norm. Hence, veganism can be part of an opinion-based identity, through people defining themselves as a group based on shared opinions (Bliuc et al., 2007).

In recent years, social psychologists have turned attention to the study of veganism and vegan identity. Recent theorizing has highlighted psychological similarities between human intergroup relations and human-animal relations (Dhont and Hodson, 2014; Dhont et al., 2014; Amiot and Bastian, 2015, 2017), and addressed human-animal relations in terms of intergroup interaction/relations (Haslam and Loughnan, 2014; Becker et al., 2019; Everett et al., 2019; Hoffarth et al., 2019; Leite et al., 2019). Previous studies mainly focus on the role of conservatism (e.g., Hodson and Earle, 2018), system justification (e.g., Caviola et al., 2019), or social dominance orientation (e.g., Dhont and Hodson, 2014), in relation to meat consumption, prejudice against vegan and vegetarians, speciesism, animal welfare concerns, and support for animal rights. However, veganism has been found to be an important part of some activist identities and social movements (e.g., Stuart et al., 2013; Vestergren et al., 2019; Judge et al., 2022). Although personality or ideology constructs such as Social Dominance Orientation (SDO), Right-Wing Authoritarianism (RWA) (see e.g., Dhont et al., 2016; Judge and Wilson, 2019), and conservatism can explain some behaviors or behavioral intentions such as meat-eating or not meat-eating that are considered as related to veganism or anti-veganism, these are not sufficient to explain veganism or vegan identity itself. An identity-focused approach, as outlined above, can offer a more comprehensive explanation in relation to becoming vegan as well as sustaining the vegan identity by going beyond the facilitators of veganism-related behaviors.

We believe that a systematic mapping review is needed to summarize the findings and identify the gaps in social psychological studies of veganism and vegan identity. These gaps are of importance to identify, not only for vegan activism and movement but also for sustainable living in relation to the environment and climate crisis.

## Method

**Figure 1:**
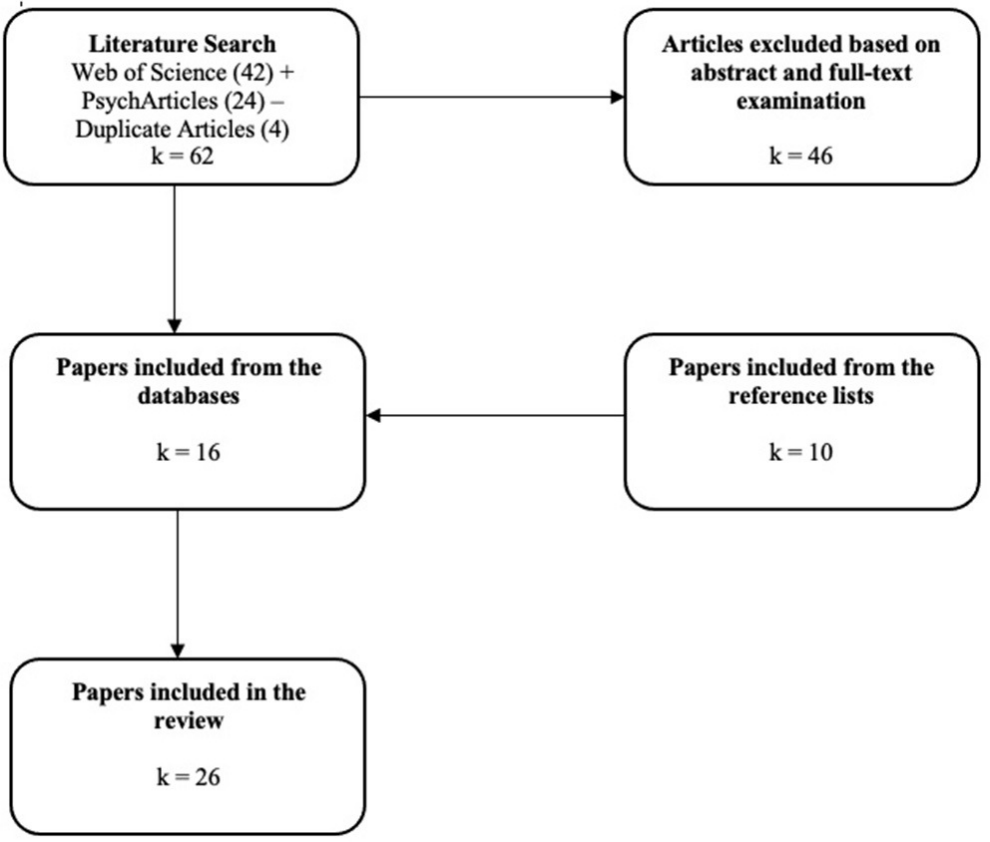
Selection process of included articles.

In accordance with PRISMA guidelines (Liberati et al., 2009) we conducted a systematic mapping review of social psychological factors related to veganism as a diet, social movement, lifestyle, or shared social identity. To be included in this review, studies had to meet the following inclusion criteria: (1) be indexed in Web of Science or PsycArticles, (2) written in English, (3) published after 2010, and (4) focused on veganism or topics related to veganism such as meat eating, speciesism, anthropocentrism, or animal right activism. The keywords used to compile the papers in our data set were “v*egan, veganism, vegetarian, vegetarianism, meat, omnivores, speciesism, animal rights, animal exploitation, animal welfare, human-animal relations*”. We purposely added search terms such as vegetarian and vegetarianism for capturing social psychological studies that merged vegetarians and vegans in their sample. Initial database searches, conducted in February 2021, using these key terms in the social psychology category of databases yielded 66 articles, from which 4 duplicates were removed. Screening of titles and abstracts was conducted independently by authors to identify articles that were relevant to the scope of the review. Where the authors did not initially agree on the articles that should be discarded, conflicts were resolved via consensus. We include records that collected data from vegans; approach veganism as an environmental, social, activist, or political identity; frames vegans as an outgroup and measure anti or pro-vegan attitudes; address vegans as a subgroup of vegetarians; explain meat-eating behaviors using social psychological perspectives and approach them as behaviors committed by group members who see vegans/vegetarians as outgroup. Records were excluded if they focus on meat-eating without emphasis on veganism-related social-psychological variables and processes such as identity, ideology (e.g., SDO, RWA, conservatism, speciesism, anthropocentrism), and intergroup relations. As seen in Figure 1, a total of 46 articles were discarded following the inclusion criteria and focus of review after abstract and full-text examination. After finalizing the first search, all included articles' reference lists were scanned. From the reference lists we included an additional 10 articles. A final data set of 26 articles were identified for analysis.

The final data set was analyzed thematically (e.g., Attride-Stirling, 2001). All papers were read thoroughly and repeatedly by both authors. While reading, notes and codes in relation to veganism and vegan identity were made. The codes were then discussed and organized into clusters based on recurring meaning in relation to veganism and vegan identity. For example, rejecting the label of vegan and vegaphobia were collated with other codes in a category of stigma and stereotyping. The categories were then reviewed and discussed between the authors to develop themes of recurring meaning within the dataset.

## Findings

Through thematically analyzing the papers in the dataset in relation to veganism and vegan identity four categories of social psychological research on veganism were created: (1) veganism as disadvantaged stigmatized identity (*n* = 10); (2) the role of ideology in attitudes toward vegans (*n* = 9); (3) the role of moral and ethical beliefs in sustaining or changing dietary preferences (*n* = 10); and (4) veganism as a social movement and vegan activism (*n* = 4). Some papers were included in more than one category based on their conceptual content (see Table 1).

**Table 1:** List of reviewed studies and their features.

**References**	**Method(s)**	**Focus**	**Data**	**Country**	**Theme(s)**
Bagci and Olgun (2019)	Quantitative: Cross-sectional survey	Vegan stigmatization, perceived discrimination, social identity needs, well-being	*N* = 350; community sample	Turkey	1
Bresnahan et al. (2016)	Quantitative: Experiments	Predictors of vegan stigma, impact of pro- and anti-vegan messages, anger, discomfort	*N*_1_ = 261, *N*_2_ = 225; student samples	*no information*	1
Butterfield et al. (2012)	Quantitative: Experiments	Anthropomorphism, support for animal welfare and rights	*N*_1_ = 42, *N*_2_ = 57; student samples	*no information*	3
Buttny and Kinefuchi (2020)	Qualitative: Critical discursive analysis to discussions	How vegans deal with their identity and problematic interaction with omnivores	7 vegan students	The US	1
Cole and Morgan (2011)	Qualitative: Discursive analysis to news	Vegan stigmatization in media	397 newspaper articles	The UK	1
Cruwys et al. (2020)	Mixed method: Qualitative and quantitative survey	Big Five, moral foundations, self-efficacy, social identification with dietary group, diet adherence	*N* = 292; community sample	*no information*	3
Davis et al. (2019)	Qualitative: Sentiment analysis and mean word counts through big data	Social identity, social movement, identity feedbacks, identity verification	9,994 YouTube comments	*multinational*	4
Dhont and Hodson (2014)	Quantitative: Cross-sectional surveys	RWA, SDO, perceived threat from non-exploitative ideologies, human supremacy belief	*N*_1_ = 260, *N*_2_ = 489; community samples	Belgium	2, 3
Dhont et al. (2014)	Quantitative: Cross-sectional surveys	SDO, ethnic prejudice and speciesist attitudes	*N* = 191; student sample	Canada	2
Dhont et al. (2016)	Quantitative: Cross-sectional surveys	Role of SDO, RWA and conservatism in speciesism and ethnic prejudice	*N*_1_ = 118, *N*_2_ = 198; student samples & *N*_3_ = 573; community sample	Belgium (Study 1) & the UK (Study 2) & the US (Study 3)	2
Earle et al. (2019)	Quantitative: Experiments	Negative attitudes toward vegans, visual reminders of meat's animal origins, empathy for animals, disgust for meat, vegan threat	*N*_1_ = 299, *N*_2_ = 280; community samples	The US	2, 3
Graça et al. (2016)	Mixed method: In-depth interviews and cross-sectional surveys	Moral disengagement of meat consumption, SDO, speciesism, human supremacy beliefs	*N*_1_ = 1013, *N*_2_ = 318; community samples	Portugal (Study 1) & the US (Study 2)	2, 3
Greenebaum (2012)	Qualitative: In-depth interviews	Contradictions of ethical vegans, impression management, vegans' presentation of self, identity performance	16 vegans	the US	1
Hodson and Earle (2018)	Quantitative: Cross-sectional survey	Reasons for adopting vegan diet, social support, conservatism	*N* = 1313; community sample	the US	2, 3
Hoffarth et al. (2019)	Quantitative: Cross-sectional surveys	SDO, conservatism, economic system justification, speciesism, attitudes toward animal welfare	*N*_1a_ = 2219, *N*_1b_ = 1500, *N*_2_ = 395; community samples	the US	2
Janssen et al. (2016)	Qualitative: In-depth interviews	Vegan motives for adherence and attitudes toward animal agriculture	329 vegans	Germany	3
Judge and Wilson (2019)	Quantitative: Cross-sectional survey	Attitudes toward vegans, RWA, SDO, dangerous worldview, competitive-jungle worldview	*N* = 1326	New Zealand	1, 2
Kalte (2021)	Quantitative: Cross-sectional survey	Vegans' political behaviors, different motives of vegans	*N* = 628 vegans; community sample	Switzerland	3, 4
Leach et al. (2020)	Quantitative: Experiments	How information about animals shifted moral beliefs about omnivores' diet and harming animals	*N*_1a_ = 241, *N*_1b_ = 213, *N*_2_ = 318, *N*_3_ = 210; student samples	The UK	3
MacInnis and Hodson (2017)	Quantitative: Cross-sectional surveys	Negative attitudes toward vegans, threat perception against vegans, bias	*N*_1_ = 278, *N*_2_ = 280, *N*_3_ = 371; community samples	the US (Studies 1 and 2) & mostly the US and Canada (Study 3)	2
Markowski and Roxburgh (2019)	Qualitative: Focus groups	Vegan stigma, behavioral distancing	Focus group discussion with 34 university students	the US	1
Potts and Parry (2010)	Qualitative: Textual examination and thematic analysis of web sources	Aggressive response of omnivore heterosexual cis-men against a particular vegan group (vegansexuals)	Comments in 12 cyberspace sources	New Zealand	1
Rosenfeld (2019)	Quantitative: Cross-sectional surveys	Different motives of vegans, disgust toward meat, dietary adherence	*N*_1_ = 361, *N*_2_ = 562; community samples	the US	3
Rothgerber (2014)	Quantitative: Cross-sectional surveys	Group vulnerability, disloyal ingroup behaviors, intergroup distinctiveness	*N*_1_ = 404, *N*_2_= 400, community samples	*no information* (Study 1) & the US (Study 2)	1
Stuart et al. (2013)	Qualitative: web sources and in-depth interviews	Multiple identity conflict, activist identity, social movement	21 editorial and commentary articles & 6 interviews	the US	1, 4
Thomas et al. (2019)	Quantitative: Cross-sectional survey	Social identification, animal right activism, politicization, radicalization	*N* = 578; community sample	the US	4

*Theme 1: vegan stigmatization, Theme 2: ideology and attitudes, Theme 3: moral and ethical beliefs in sustained and changed diet, Theme 4: social movement and activism*.

### Vegans as a Disadvantaged and/or Stigmatized Group

Many studies on veganism or vegans within the social psychological discipline use a critical discursive framework to focus on vegans as a disadvantaged stigmatized group and seek the predictors of vegan stigma (e.g., Rothgerber, 2014; Bresnahan et al., 2016; Markowski and Roxburgh, 2019). For instance, through a discursive analysis that critically examined *vegaphobia* in the UK newspapers it was demonstrated that vegans were stigmatized and stereotyped as unrealistic sentimentalists, fanatics or extremists (Cole and Morgan, 2011). Similarly, Potts and Parry (2010) focused on online comments in digital media and identified aggression toward particular vegan groups, labeled as *vegansexuals* (vegans who have romantic or sexual relationships with only vegan people), by heterosexual omnivore cis-men. In Potts and Parry's study, vegans were found to be stereotyped in a negative way and labeled as deviants and bigots. The authors suggest that the relationship between meat-eating and masculinity in western societies could be a potential reason for cis-men's aggressive response to refusal of the meat culture. The masculinity, or lack thereof in relation to veganism could be suggested to stem from a social identity where vegans are seen as “soft and caring” as a consequence of the identity framework of non-harm.

While some studies focus on the predictors of vegan stigma, others have focused on how vegans perceive stigmatization and the consequences of stigmatization. Bagci and Olgun (2019) examined how vegans and vegetarians in Turkey perceive stigmatization and whether social identity needs (esteem, meaning, belonging, efficacy, distinctiveness, and continuity) were associated with perceived discrimination of vegans. They showed that satisfaction of esteem and meaning needs were the most correlated variables with perceived discrimination (Bagci and Olgun, 2019). Furthermore, through a discursive analysis, focusing on stigmatized vegans' problematic interaction with omnivores, it was found that vegans experienced several ideological dilemmas in relation to their different identity manifestation or performance such as veganism as choice of diet, for environmental reasons or ethical considerations (Buttny and Kinefuchi, 2020). Stuart et al. (2013) demonstrated effects of vegan stigmatization in an activist group where members have multiple identities. Although most of the Sea Shepherd Conservation Society members position themselves as radical activists, some of them rejected being labeled as vegan to avoid being considered as “hardcore vegan” which implies an inflexible ideological position, and the organization should keep a distance from this position. Moreover, Markowski and Roxburgh (2019) showed that stigmatization of vegans also has negative impacts for omnivores as it can inhibit dietary shifts toward veganism due to the negative label. Related to the perception of stigmatization and its consequences are the attitudes toward vegans, and especially the role that ideology plays in creating and maintaining the attitudes.

### The Role of Ideology in Attitudes Toward Vegans

There are an increasing number of studies in social psychology focusing on the role of ideology-related variables such as right-wing authoritarianism (RWA), social dominance orientation (SDO), system justification, and political conservatism in human-animal relations in relation to attitudes toward vegans and vegetarians. Many of these studies argue that ideological variables that shape human-human relations, and prejudice or discrimination against human outgroups, also predict human-animal relations. Hence, they seek common ideological roots of speciesism and negative human outgroup attitudes (e.g., Dhont et al., 2014). Beyond the commonalities of human-human and human-animal relations, these studies position ideological variables at the core of negative attitudes toward stigmatized dietary and/or political groups such as vegans.

SDO and RWA as the most prominent right-wing ideological variables in social psychology have been tested as predictors of the speciesist attitudes. Both SDO and RWA have been found to predict negative attitudes toward vegans (Judge and Wilson, 2019). However, Dhont et al. (2014, 2016) found that SDO, more than RWA, was associated with speciesism. Moreover, they showed that both SDO and RWA were related to perceived threats against vegans and vegetarians. Perceived threat against non-meat eating groups was also found to mediate the relationships between SDO-RWA and meat consumption (Dhont and Hodson, 2014). Furthermore, beliefs in human superiority mediated the relationship between SDO and meat consumption. Similarly, MacInnis and Hodson (2017) demonstrated that omnivores have high levels of prejudice against vegans, and this prejudice was much higher among those scoring high in right-wing ideologies. They also found that omnivores have more negative attitudes toward vegans who are motivated by animal rights or environmental concerns than those motivated by health concerns. Relatedly, Hoffarth et al. (2019) found that political conservatism, in addition to SDO, was associated with greater endorsement of speciesism through economic system justification. As previous studies demonstrate, ideology is a strong predictor of attitudes toward vegans. When the perspective is turned and instead focuses solely on the consumer's dietary change or adherence, the main predictors identified previously are conceptualized as moral and ethical beliefs.

### The Role of Moral and Ethical Beliefs in Changing or Sustaining (Vegan) Diet

Research on changing or sustaining meat-eating and plant-based diets mainly revolve around two predictors: moral and ethical beliefs. In their study of predictors of dietary adherence, Cruwys et al. (2020) found that the most frequently occurring facilitator for sustained diet was ethical/moral concerns (51.6%). Furthermore, in relation to veganism participants described their vegan diet as an “ethical way of life” (p.7). References to “way of life” and “lifestyle” in the dataset could further indicate the perception and incorporation of social identity as a vegan, or veganism as part of identity content.

In addressing changed dietary preferences in human-animal relations studies, there is an emphasis on the participants' morality and moral beliefs. For instance, Leach et al. (2020) examined whether receiving information about animals' traits and behaviors change moral beliefs about eating meat. They found that information that animals can feel nostalgia (i.e., secondary emotion) and others' suffering (i.e., empathy) as well as animals' capacity to feel pain affected individuals' moral beliefs about harming animals. Correspondingly, Butterfield et al. (2012) found that attributing human characteristics to animals (i.e., anthropomorphism) was associated with positive attitudes toward vegans. Graça et al. (2016) showed that frequency of eating meat was strongly associated with moral disengagement of meat consumption. Moreover, individuals' moral variables such as level of empathy, moral identity, and moral emotions was related to moral disengagement of meat consumption. Similarly, Earle et al. (2019) found that increased empathy for animals mediate the relationship between visual reminders of meat's animal origins and decreased meat consumption. Hence, previous research demonstrates a strong case for the role of morality in changing to a more plant-based diet. When it comes to sustaining that dietary preference, most emphasize the role of ethical beliefs.

More than 70% of surveyed vegans consider animal rights the most important reason to being vegan (Kalte, 2021). Hodson and Earle (2018) explored reasons for lapsing back to meat eating after being vegan and found that former vegans, compared to current vegans, were less likely to be motivated by social justice along with scoring higher in conservatism. Rosenfeld (2019) focused on the ethical-health dichotomy in veganism and compared dietary goal orientation in terms of dietary adherence. Rosenfeld found that animal rights motivated vegans and vegetarians displayed stronger adherence than environmentally and health motivated vegans and vegetarians. Moreover, higher disgust toward meat among animal rights motivated vegans and vegetarians was associated with their stronger adherence. The motives for sustained diet are important for wider consumer habits and thereby extends beyond what is present on the dinner table. For example, Janssen et al. (2016) showed that ethical and self-oriented vegan consumers have different attitudes toward animal agriculture. They found that vegan consumers who did not refer to ethical concerns such as animal rights motives had more positive attitudes toward animal agriculture. Even though ideology is emphasized in attitudes toward vegans and veganism, and moral and ethical beliefs demonstrated as crucial in changing and sustaining diet there are few studies that go beyond the individual level of veganism. In the next, and last, category we outline studies that include a dimension of collectivism in the form of social movements or veganism as activism.

### Veganism as a Social Movement and Vegan Activism

One of the most neglected areas of social psychological studies on vegans is activism and social movement aspects of veganism. While seeking the answer to whether veganism is an individualized form of political participation Kalte (2021) showed that a majority of vegans are politically motivated. However, when focussing on social movements, veganism as activism, and collective identity processes there is very sparse research. Stuart et al. (2013) identified in their interviews with anti-Whaling activists that veganism can, but does not necessarily need to, be part of activist identity content. Thomas et al. (2019), following the social identity approach, classified animal right activists by using latent profile analysis. They identified three animal rights activist groups: omnivores, lifestyle activists, and vegetarian radical groups. In their study, Thomas et al. (2019) demonstrated that participants who had higher vegan identification tended to be more committed to radical actions. Finally, Davis et al. (2019) argue that veganism is a social movement identity by using qualitative analysis of YouTube comments and showing that non-verifying identity feedbacks elicit negative emotional response among vegans. Through sentiment analysis with a qualitative element they demonstrate how distress is created when the content is not aligned with the perceived collective vegan identity, and how positive emotions increase when the content and collective identity are aligned.

Although previous research makes important contributions to insights about human-animal relations and attitudes toward vegans, veganism and vegan identity is not addressed comprehensively within social psychology. Consequently, based on the results from our systematic mapping review, we argue that there are several issues that need to be addressed in how vegans are viewed in the social psychological literature. These issues are mainly in relation to the identity content and context of vegans and veganism.

## Discussion

In our systematic mapping review we created four categories of study focus and content in relation to veganism and vegan identity: stigmatization, ideology and attitudes toward vegans, moral and ethical beliefs in changing and sustaining diet, and veganism as social movement and vegan activism. Based on the reviewed literature we argue that there are four crucial issues and gaps needed to discuss: (1) merging all non-meat eaters, (2) reduction of veganism to dietary or life-style choices neglecting the politicized content and movement, (3) lack of social psychological processes of emergence and endurance of vegan identity, and (4) decontextualization of vegan identity and lack of cultural factors.

### Merging all Non-Meat Eaters

We argue that vegetarian and vegan identities are distinct identities. This argument follows theorizing by MacInnis and Hodson (2021) who found that, for example, vegans often prefer vegans over vegetarians. Further adding to the need for a distinction between vegans and vegetarians MacInnis and Hodson (2021) found that both groups had more positive experiences within their own group (and more negative with the outgroup). Furthermore, vegan identity is often politicized whereas vegetarian identity may or may not be politicized. For example, vegans are often politically active (Stuart et al., 2013; Kalte, 2021) and radical animal rights activists are often vegan (Stuart et al., 2013). Kalte (2021) found that 89% of the vegans reported political reasons for being vegan. Similarly, Cruwys et al. (2020) found that vegans often understood their dietary choices in terms of social and political contexts, were vegans (80.5%) more often than vegetarians (46.7%) emphasized moral and ethical reason for their sustained diet. Even though only a small difference, vegans (9.8%) referred to a shared identity more often than vegetarians (8.9%) (Cruwys et al., 2020). Moreover, Markowski and Roxburgh (2019) demonstrated that vegetarians and omnivores often shared negative perceptions of vegans and veganism, further highlighting the need for differentiation between non-meat eaters. Hence, even though similar, there are differences between vegans and vegetarians in terms of politicized content and identification which suggests that they should be studied as distinct groups.

Human-animal relation research lacks focus on the role of shared social identity in human-animal relations and veganism. To our knowledge, there are only three studies that addressed veganism/vegetarianism with insights from the social identity approach (i.e., Thomas et al., 2019; Cruwys et al., 2020; Judge et al., 2022). Thomas et al. (2019) identified three profiles (ambivalent omnivores, life-style choice activists, and vegetarian radicals) who all engaged in animal welfare actions. However, the authors did not address vegan identity as a distinct identity, instead, it was conceptualized as a vegetarian lifestyle activist group or radical vegetarian profile (including both vegan and vegetarians). Cruwys et al. (2020) differentiated between vegans and vegetarians; however, their focus was on adherence to diet rather than the dimensions of such identities. Judge et al. (2022) approach veganism as an activist identity, and emphasize the action based framework for the identity.

We suggest that vegan identity, as different from vegetarian identity, should be addressed as a (disadvantaged) politicized social identity. Related to the neglect of differentiating between non-meat-eating identities, there is also a neglect of the content of vegan identity often reducing it to dietary or lifestyle choice.

### Reduction of Veganism Into Dietary or Lifestyle Choices, Neglecting the Politicized Content and Movement

Veganism can be argued to be more than a lifestyle, it can be seen as a feature of a social movement standing against the exploitation of animals (including humans) and environment (e.g., Vestergren et al., 2018, 2019). Through their choice of diet activists can display and perform their activist identity (e.g., Vestergren et al., 2019). Activist Michael Pollan emphasize how people make political acts by using their fork, and states “The wonderful thing about food is that you get three votes a day. Every one of them has the potential to change the world” (Nourish, 2020). Hence, veganism, as a tool for political motives and behaviors, can be seen as a vehicle for societal change (Kalte, 2021).

We acknowledged that human-animal relations are complex, precarious, and paradoxical as some animals are loved family members and others slaughtered. However, there is a need to focus on how people understand these paradoxical behaviors. For example, Leach et al. (2020) suggest that people change their moral beliefs about animals as food or friends based on information about the animal's ability. They highlight animal abilities such as feeling secondary emotions, understanding morality, capacity for empathy, forming social bonds and experiencing negative emotions as key dimensions for why participants would not consider them as food (Leach et al., 2020). Interestingly the authors did not find type of diet (vegan, vegetarian, pescatarian, omnivore) to qualify for main effects in any of their studies. Consequently, regardless of diet, the moral judgements were similar throughout the data set. Hence, the decision of whether an animal is food or not was not dependent on the current diet. This could indicate that being vegan contains more than just adhering to dietary choices or animal characteristics. Therefore, vegans, as a political group who stand against animal and environmental exploitation, should be more than objects of social psychological studies.

Vegans, and the motives for veganism, can be organized under three types: health (e.g., Radnitz et al., 2015; Cramer et al., 2017), animal rights (e.g., Greenebaum, 2012), and environment (e.g., Janssen et al., 2016). Greenebaum (2012) uses the terms “ethical” vegans and “environmental” vegans to differentiate between animal-rights and environmental vegans. The division of vegans is based upon the behaviors and attitudes they might hold. For example, environmental vegans might prefer second-hand leather over PVC, whereas ethical vegans might condone the use of soy (Greenebaum, 2012). Hence, the content of the shared identity, and thereby legitimate action, can vary between different types of vegans. In the case of health vegans, the dietary choices are assumed to be more related to self-interest rather than a shared political identity. The division into these types of vegans in previous literature mainly consists of asking participants about their main motive for veganism. However, by doing so there is a risk of neglecting similarities and overlaps. For example, when the question targeted main motive, Kalte (2021) found that 71% stated animal welfare and only 12% environment as their main reason for being vegan. However, when asking for important factors of their veganism (without having to choose only one) 78% stated that environmental concerns was an important factor for their veganism (see also Janssen et al., 2016).

Consequently, previous research can be seen as reductionist in terms of identity content as veganism is often referred to in the context of animal-human relations. However, reduction of animal products has been emphasized as an important element in fighting the climate crisis (Peta, 2015). Therefore, there is a need to be more inclusive in the theorizing around veganism and go beyond the animal related foundation to explore vegan identity and all its dimensions. Addressing the social and political dimensions of the identity content includes going beyond treating veganism as an individual choice where the shared ideological, moral, or ethical content of the identity becomes shadowed by the more practical individual choice of diet or lifestyle. A few previous studies have identified consumption choices as content of a salient social identity (e.g., Stuart et al., 2013; Vestergren et al., 2018, 2019). Vegan identity might be included in both animal rights and environmental rights activist identity and the intersectionality of these needs addressing. To our knowledge, no studies have yet sought to explore the distinction or inclusion of these two dimensions in relation to a wider vegan identity. Examining vegans' construal of what it means to be vegan, by exploring similarities and differences between animal rights vegans and environmental vegans and discussing the intersectionality between animal-environmental dimensions/identities is needed.

Kurz et al. (2020) argue that vegan identity can be seen as a moralized-minority-practice identity (MMP) and thereby has further implications in terms of accepted or normative behaviors. In comparing newspaper accounts of meat-eaters and vegans, Cole and Morgan (2011) found that vegans were portrayed in a derogatory manner, described as hostile extremists, ascetics, fad, and oversensitive, and were ridiculed for promoting something that was portrayed as impossible to sustain. Several additional studies have emphasized stigmatization toward vegans (Potts and Parry, 2010; Wright, 2015; MacInnis and Hodson, 2017; Markowski and Roxburgh, 2019) and strategies to cope with the stereotypical attributes (Buttny and Kinefuchi, 2020; Schwartz, 2020). For example, Israeli vegans adopted strategies to de-stigmatize veganism such as adhering to masculine features by posting pictures reflecting strength and muscles, reporting male behaviors such as barbequing, or ridiculing left-wing ideology to compensate for their veganism and navigate different identities (Schwartz, 2020). Similarly, Buttny and Kinefuchi (2020) suggests that vegans might use the term “plant-based” instead of vegan to avoid the negative stigma and highlight the ideological dilemma vegans face in terms of when and how to communicate their beliefs and attitudes. By developing strategies to deal with the ideological dilemma vegans acknowledge that their identity is stigmatized and goes against the normative mainstream culture (Buttny and Kinefuchi, 2020). Moreover, prejudice against vegans has been demonstrated to be stronger when ideological dimensions are involved. For example, prejudice views of being vegan for moral and ethical reasons, in relation to animal rights and environment, were higher than toward people who were vegan for health reasons (MacInnis and Hodson, 2017). To sum up, in relation to previous research it is suggested that reducing veganism to an individual lifestyle or dietary choice neglects the underlying social, ideological, and political dimensions of the identity. In addressing the content of the shared identity, it is also important to address the processes of emergence and endurance of the identity.

### Processes of Emergence and Endurance of a Vegan Identity

Social psychological studies of veganism and vegan identity have mostly overlooked addressing factors underlying the process of becoming vegan. In general, contemporary research emphasizes questions in relation to single variables predicting justifying eating meat (or not), and why some people endorse speciesism more than others (Dhont and Hodson, 2014; Hoffarth et al., 2019; Leach et al., 2020). We propose that veganism can be part of a politicized social identity, as well as it can emerge through intragroup interaction with other vegans or activists (see also Drury and Reicher, 2000; Vestergren et al., 2019). In a study of environmental activists, Vestergren et al. (2019) found that some participants became vegan through their participation in collective action. The participants explained this change because of change in the way they viewed themselves and their social world, specifically, the change in consumption was related to a change in perceived intergroup and intragroup relations (Vestergren et al., 2019). Similarly, Cherry (2015) found that participants in the punk movement changed their diet to become vegan. The shift in diet was suggested to emerge through a shift in identity, lifestyle, emerged through intragroup interaction. The reconstruction of the identity was related to moral and ethical issues and what the punk movement stood for. Therefore, the emergence of veganism was related to a reconstruction of identity content. Social psychological research should explore the reasoning behind becoming vegan and factors motivating the change in behavior and beliefs. Hence, research needs to explore what the processes of emerging vegan identity are, ingroup norms of movements where veganism is common, types of identity processes involved and more specifically, how people navigate the status quo and politicized vegan identity.

In addition to the lack of accounts of social psychological processes of emergence, there is also a lack of detailed processes of endurance of vegan identity. Previous studies demonstrate that different motives of being vegan affects whether the diet is sustained or not (e.g., Moore et al., 2015; Cruwys et al., 2020). However, these studies mainly focus on a general concept such as morality. In addition to the moral and ethical animal-rights factor affecting endurance, other political dimensions have been identified that should be addressed and explored on a deeper level along with the meaning of morality and ethical motives of sustained diet. For example, the connection between diet and ideology was demonstrated in relation to sustained diet (Hodson and Earle, 2018). Hodson and Earle (2018) found that conservative vegans relapsed (resume meat consumption) at higher rates than vegans who held a more liberal ideology (Hodson and Earle, 2018). As previous research has identified that different motives to veganism affect adherence, these need to be explored further to identify what it is more specifically about the various motives. We argue that being motivated by ideological or ethical reasons also contains a dimension of social identity and group membership that reasons related to individual health do not. Perceiving to be part of a social group, sharing a social identity, also brings with it expected support and social networks.

The importance of shared identity and social support has been identified in relation to sustained veganism. However, in most studies, the factors used to explain sustained diet excludes the social dimension, or if included (e.g., Hodson and Earle, 2018; Cruwys et al., 2020) there is no further theorizing what it is about the social that facilitates endurance. For example, Cruwys et al., (2020) found that participants who identified with their dietary group also were more likely to sustain their diet. Similarly, Hodson and Earle (2018) found that their participants who lapsed back from veganism to meat-eating lacked social support in relation to their veganism. However, none of the studies go beyond the variable to try to explain what it is about the social that facilitates adherence to veganism. We argue that intragroup relations/interaction can facilitate the endurance of identity content, in this case actions and beliefs related to veganism (see Vestergren et al., 2018). Related to the social dimension is identity performance, if you are in a social space where you can perform your identity content, for example eating vegan or discuss legitimate behaviors with like-minded, you get to enact your identity which could further facilitate the endurance (see Vignoles et al., 2006, 2011; Klein et al., 2007). Previous studies have emphasized the importance of social networks, to exchange knowledge and resources, for endurance of environmental identity (Kennedy, 2011; Vestergren et al., 2018). Similar to Cherry (2006, 2015), we argue that veganism can be a social identity, and for this identity to endure the social relations informing it needs to be sustained. However, there is a lack of research exploring what it is specifically about these social networks, why and how they facilitate a sustained vegan identity or lifestyle. Hence, we need an account of what it is about the perceived social interaction that facilitates a vegan identity to be sustained. In addressing the social interaction that vegan identity emerges and endures, it is also crucial to include the cultural context that the interactions occurred.

### Decontextualization

The fourth identified gap in the existing literature relates to decontextualization of the human-animal relations, and related intergroup behaviors such as animal liberation movements or veganism. If human-animal relations should be addressed as an intergroup relation (see Amiot and Bastian, 2015), then we should not neglect the effect of cultural contexts in intergroup relations (see bimodal relationships; Klein et al., 2007). Shared social identity provides definitions of possible conduct and enables people to act collectively in normative ways according to ingroup norms (see Drury and Reicher, 2000). However, it is important to acknowledge that enactment of ingroup norms also takes place in intergroup contexts where ingroups and outgroups might have different values and perceptions of these norms. These intergroup contexts are often created by the dynamic actions of other groups such as political and religious authorities, anti-vegans, meat-eaters, or third parties. In other words, there is no universal or singular performance for vegan identity across cultures, the performance will depend on the structures of the social interactive and cultural context they are placed in.

Previous research on psychological change through participation in social movements have identified both intergroup and intragroup as crucial for psychological changes to emerge and endure (e.g., Vestergren et al., 2018, 2019). Accordingly, we believe that interaction and cultural beliefs may deeply affect the factors that motivate people to become vegan, stay vegan, and how vegan and animal liberation movements escalate in specific intergroup contexts. For instance, one in ten people in Sweden identifies as vegetarian or vegan (Molloy, 2014). In 2018 two percent (~202,400) of Sweden's population (2018: 10.12 million) reported being vegan (Statista, 2020a). This was a decrease compared to four percent in 2015. Contrary to Sweden, other countries have seen a large increase in veganism. In the UK, the vegan population grew from 150,000 in 2014 to over 600,000 in 2018 (Statista., 2020b). Moreover, the UK launched more vegan products than any nation (Mintel, 2019). Culturally, other countries face even more complex dimensions of veganism than Sweden and the UK. For example, being Muslim may add an even more complex relationship with veganism and vegan identity. Meat consumption, through sacrifice, is one of the five main religious duties of Muslims. Hence, for many Muslims there is a need to navigate their vegan and Muslim identity. Nevertheless, the vegan movement is increasing in some Muslim countries too, such as Turkey (Rasmussen, 2017) and vegans are finding strategies to cope with complexities such as veganism and Eid. Hence, vegan identity, just like other shared social identities, are affected by social locations and positions and require various social identity performances across different times, cultures, and contexts (see Drury et al., 2012). Moreover, political meta-factors such as political openness (Saavedra and Drury, 2019), trust (van Stekelenburg and Klandermans, 2018), procedural justice (Gerber et al., 2018), presence of violent repression (Ayanian et al., 2021), political regime type (Regan and Henderson, 2002) have important impacts on diversity of social movements, in turn, emergence, endurance, change and performance of shared social identity across cultures. We suggest that underlying factors of politicized vegan identity formation and performance might have different cultural patterns although the main ideological and ethical reasons might be the same. Alongside the processes of emergence and endurance of vegan identity, we argue that there is a need to focus on the intersectionality of vegan identity, not only in terms of ideology and politics, but also in terms of culture and context.

## Conclusion

Our aim with this paper was to gather and compare the studies of veganism and vegan identity. Through a systematic mapping review of the literature on veganism and vegan identity within the social psychological field of research we identified a data set of 26 papers published between 2010 and 2021. This review is not intended to gather and outline all studies on veganism and vegan identity, as there are studies within different disciplines such as sociology and nutrition. This review constitutes a comprehensive account of social psychological studies identified using general key terms related to veganism. Through analyzing the papers thematically, we created four categories of study focus and content: stigmatization of vegans, role of ideology in negative attitudes toward vegans, role of moral and ethical beliefs in changing or sustaining veganism, and veganism as a social movement and vegan activism, and conclude that there are four main gaps in the literature that needs addressing in future studies: merging all non-meat eaters, reduction of veganism to dietary choices or lifestyle, lack of processes underlying emergence and endurance of veganism, and decontextualization of vegan identity. We argue that filling these gaps are fundamental on several levels. Firstly, in addressing the climate crisis and fighting it we need to focus on both individual and collective solutions. Providing research of for example sustainable living (incl. diet) would be facilitated by understanding the wider identity content and context to facilitate individual and collective action. Secondly, in an increasingly “unhealthy” world there is a need to focus on more healthy living in terms of individual diets. Dietary recommendations and research should include processes of emergence and endurance of such diets, and how the importance of a social dimension needs to be included. Thirdly, identities are not stable, they vary depending on the social context and are influenced by the intersectionality of other identities. These elements, variability and intersectionality, need to be further explored to advance not only theorizing around shared identities but also in terms of fighting prejudice, climate crisis, inequalities. Finally, throughout our systematic mapping review, as well as our own theorization (and recent literature e.g., Judge et al., 2022) there is still an elephant present in the room. The importance of understanding veganism in a social and political perspective is clear, as well as the relation to a minoritised, politicized, opinion-based identity. However, what still is not clear, and needs to be addressed further, is whether vegan identity is the framework for a shared identity or part of the identity content in related social identities (e.g., climate change activist identity). Only by including these elements can theories, structures, and policies become inclusive and general beyond the individualized core to facilitate for the much needed social and behavioral changes addressed by the International Panel of Climate Change 2022.

## Data Availability Statement

The original contributions presented in the study are included in the article/supplementary material, further inquiries can be directed to the corresponding author/s.

## Author Contributions

Both authors listed have made a substantial, direct, and intellectual contribution to the work and approved it for publication.

## Conflict of Interest

The authors declare that the research was conducted in the absence of any commercial or financial relationships that could be construed as a potential conflict of interest.

## Publisher's Note

All claims expressed in this article are solely those of the authors and do not necessarily represent those of their affiliated organizations, or those of the publisher, the editors and the reviewers. Any product that may be evaluated in this article, or claim that may be made by its manufacturer, is not guaranteed or endorsed by the publisher.
